# Correction: Raman Subrahmanyam, et al. On the Road to Biopolymer Aerogels—Dealing with the Solvent. *Gels* 2015, *1*, 291–313

**DOI:** 10.3390/gels2030021

**Published:** 2016-08-09

**Authors:** Raman Subrahmanyam, Pavel Gurikov, Paul Dieringer, Miaotian Sun, Irina Smirnova

**Affiliations:** Institute of Thermal Separation Processes, Hamburg University of Technology, Eißendorfer Straße 38, 21073 Hamburg, Germany; pavel.gurikov@tuhh.de (P.G.); dieringer.paul@gmail.com (P.D.); miaotian.sun@tuhh.de (M.S.); irina.smirnova@tuhh.de (I.S.)

The authors wish to make the following correction to their paper [1]. Equations (10) and (11) are empirically derived curve fitting equations from a calibration plot (Figure S1 in [1]) that were computed to calculate the concentration (wt %) of a DMSO–water mixture in dependence of its density. However, the given equations contain rounded values for the computed polynomial factors that yield incorrect concentration values. Therefore, following adjustments should be made to the equation in the paper (Equations (10) and (11)), so that correct calculations are possible for researchers accessing the paper. All calculations in this paper were undertaken using the accurate values. Therefore, all data presented are valid. Alternatively, the readers can also refer to the calibration plot (Figure S1 in [1]) in the supplementary material for density–concentration (wt %) correlations.

c(ρ) = a_6_ · ρ^6^ + a_5_ · ρ^5^ + a_4_ · ρ^4^ + a_3_ · ρ^3^ + a_2_ · ρ^2^ + a_1_ · ρ + a_0_,  for c < 82 % (*R*^2^ = 0.9990) (10)

where
a_6_1.255055740242850 × 10^9^a_5_−7.879390173815560 × 10^9^a_4_2.06071729327642 × 10^10^a_3_−2.87375597190484 × 10^10^a_2_2.25377368737041 × 10^10^a_1_−9.42486927160501 × 10^9^a_0_1.64185361883457 × 10^9^

c(ρ) = b_3_· ρ^3^ + b_2_· ρ^2^ + b_1_· ρ + b_0_,  for c ≥ 88% (*R*^2^ = 0.9694) (11)

where
b_3_−8.79975201417487 × 10^8^b_2_2.90837735148691 × 10^9^b_1_−3.20412910529304 × 10^9^b_0_1.17665251692243 × 10^9^

We apologize for any inconvenience caused. The manuscript will be updated and the original will remain available on the article webpage.

## References

[1] Subrahmanyam R., Gurikov P., Dieringer P., Sun M., Smirnova I. (2015). On the Road to Biopolymer Aerogels—Dealing with the Solvent. Gels.

