# Supplementary material for: On the Road to Biopolymer Aerogels—Dealing with the Solvent
Source: Gels. 2015 Dec 21;1(2):291–313. doi: 10.3390/gels1020291 (PMC6318595; doi:10.3390/gels1020291)
Supplement: Supplementary File 1 [file gels-01-00291-s001.pdf]

Supplementary Information

Table S1. Volumetric yield (Y_v) of the gels after various process steps (ΔC = 30 wt % ethanol).

Gel Composition	Before Gelation %	After Gelation %	Swelling %	SE (Step 1) %	SE (Step 2) %	SE (Step 3) %	SE (Step 4) %	Supercritical CO_2 Drying %
I	100	78.3 ± 0.8	97.4 ± 1.8	81.5 ± 2.4	69.4 ± 1.3	63.8 ± 1.0	59.8 ± 0.5	41.1 ± 1.8
II	100	76.1 ± 0.7	88.8 ± 0.8	77.3 ± 0.7	38.6 ± 0.6	64.0 ± 0.9	60.6 ± 0.9	42.6 ± 1.3
III	100	72.4 ± 0.4	88.5 ± 0.8	68.4 ± 2.0	49.9 ± 0.9	41.3 ± 0.6	37.5 ± 0.5	19.0 ± 1.1
IV	100	54.5 ± 0.4	57.0 ± 0.7	49.8 ± 0.2	46.2 ± 0.2	43.7 ± 0.3	41.6 ± 0.2	26.4 ± 0.6
V	100	65.6 ± 0.5	90.7 ± 1.4	65.6 ± 1.3	41.1 ± 0.7	31.2 ± 0.2	27.6 ± 0.1	12.1 ± 1.5
VI	100	50.1 ± 0.9	49.6 ± 1.0	40.1 ± 0.2	36.7 ± 0.1	34.3 ± 0.2	32.1 ± 0.1	12.4 ± 0.3

Table S2. Volumetric yield (Y_v) of the gels after various process steps (ΔC = 50 wt % ethanol).

Gel Composition	Before Gelation %	After Gelation %	Swelling %	SE (Step 1) %	SE (Step 2) %	SE (Step 3) %	Supercritical CO_2 Drying %
I	100	78.9 ± 0.8	95.2 ± 1.6	66.3 ± 1.3	55.1 ± 0.4	53.6 ± 0.5	38.1 ± 1.9
II	100	76.8 ± 0.3	90.6 ± 1.3	66.3 ± 1.0	56.2 ± 0.3	54.8 ± 0.3	39.4 ± 0.8
III	100	72.2 ± 0.4	89.5 ± 1.2	44.4 ± 0.9	30.9 ± 0.1	29.2 ± 0.2	15.5 ± 0.4
IV	100	54.0 ± 0.7	57.0 ± 0.5	45.5 ± 0.2	40.6 ± 0.2	38.9 ± 0.4	21.9 ± 0.5
V	100	66.3 ± 0.4	90.1 ± 0.9	33.1 ± 1.2	20.8 ± 0.3	19.6 ± 0.3	8.7 ± 0.9
VI	100	50.2 ± 0.7	48.7 ± 0.3	34.2 ± 0.2	30.2 ± 0.1	29.0 ± 0.3	12.5 ± 0.9

Table S3. Volumetric yield (Y_v) of the gels after various process steps (ΔC = 30 wt % DMSO).

Gel Composition	Before Gelation %	After Gelation %	Swelling %	SE (Step 1) %	SE (Step 2) %	SE (Step 3) %	SE (Step 4) %	Supercritical CO_2 Drying %
I	100	80.7 ± 0.5	92.4 ± 1.0	87.9 ± 1.8	80.9 ± 2.1	76.7 ± 2.1	73.3 ± 1.9	35.4 ± 4.1
II	100	79.0 ± 0.7	91.7 ± 1.3	87.2 ± 1.0	81.4 ± 1.0	77.9 ± 0.8	74.6 ± 0.7	40.1 ± 0.8
III	100	76.1 ± 0.4	95.5 ± 2.0	93.7 ± 3.1	82.3 ± 1.2	70.6 ± 0.6	61.3 ± 0.7	19.4 ± 0.6
IV	100	52.2 ± 0.4	58.7 ± 1.0	55.8 ± 0.3	53.6 ± 0.3	52.0 ± 0.4	50.0 ± 0.1	24.3 ± 1.1
V	100	69.7 ± 0.6	88.0 ± 1.1	55.7 ± 0.3	50.1 ± 0.1	45.7 ± 0.1	41.7 ± 0.1	4.3 ± 0.9
VI	100	51.3 ± 0.6	50.0 ± 0.7	46.3 ± 0.7	43.4 ± 0.3	41.5 ± 0.3	39.3 ± 0.5	14.9 ± 0.5

Table S4. Volumetric yield (Y_v) of the gels after various process steps (ΔC = 50 wt % DMSO).

Gel Composition	Before Gelation %	After Gelation %	Swelling %	SE (Step 1) %	SE (Step 2) %	SE (Step 3) %	Supercritical CO_2 Drying %
I	100	80.0 ± 0.5	92.6 ± 1.2	77.1 ± 0.7	70.1 ± 1.0	68.5 ± 1.0	37.2 ± 2.1
II	100	79.2 ± 0.2	91.3 ± 0.5	76.8 ± 0.6	69.4 ± 0.6	67.6 ± 0.7	35.7 ± 0.8
III	100	76.1 ± 0.7	91.2 ± 0.6	67.1 ± 0.6	50.5 ± 0.9	46.9 ± 0.6	15.8 ± 0.8
IV	100	52.5 ± 0.1	58.2 ± 0.8	52.0 ± 0.3	48.6 ± 0.3	48.2 ± 1.4	25.9 ± 0.3
V	100	70.0 ± 0.4	88.5 ± 1.2	49.2 ± 0.3	42.7 ± 0.3	40.2 ± 0.3	4.5 ± 0.2
VI	100	51.3 ± 0.6	50.4 ± 0.2	42.4 ± 0.2	39.0 ± 0.2	37.2 ± 0.3	13.9 ± 0.4

Table S5. Calculated qe (g_{sol}/g_{gel}) values for various gel compositions for 30% ethanol concentration gradient.

Gel Composition	I	II	III	IV	V	VI
Step 1	0.296 ± 0.003	0.296 ± 0.003	0.309 ± 0.005	0.308 ± 0.009	0.307 ± 0.005	0.308 ± 0.003
Step 2	0.240 ± 0.007	0.251 ± 0.003	0.214 ± 0.001	0.276 ± 0.005	0.148 ± 0.025	0.280 ± 0.006
Step 3	0.226 ± 0.011	0.236 ± 0.002	0.191 ± 0.016	0.262 ± 0.011	0.148 ± 0.010	0.156 ± 0.007

Table S6. Calculated k_2 (× 10^2) ($g_{sol}/g_{gel}\cdot min^{-1}$) values for various gel compositions for 30% ethanol concentration gradient.

Gel Composition	I	II	III	IV	V	VI
Step 1	3.440 ± 0.716	3.373 ± 0.325	2.779 ± 0.190	4.851 ± 0.724	3.128 ± 0.300	6.013 ± 0.217
Step 2	2.679 ± 0.301	3.247 ± 0.401	2.980 ± 0.089	3.819 ± 0.285	3.706 ± 0.781	4.325 ± 0.361
Step 3	3.429 ± 0.437	3.141 ± 0.348	3.300 ± 0.441	3.729 ± 0.370	4.864 ± 0.575	4.617 ± 1.073

Table S7. Calculated h (× 10^3) ($g_{sol}/g_{gel}\cdot min^{-1}$) values for various gel compositions for 30% ethanol concentration gradient.

Gel Composition	I	II	III	IV	V	VI
Step 1	2.778 ± 0.252	2.948 ± 0.267	2.656 ± 0.160	4.589 ± 0.486	2.934 ± 0.195	5.709 ± 0.253
Step 2	1.534 ± 0.094	2.056 ± 0.300	1.366 ± 0.049	2.898 ± 0.170	0.789 ± 0.118	3.380 ± 0.149
Step 3	1.744 ± 0.166	1.749 ± 0.207	1.186 ± 0.038	2.542 ± 0.155	1.067 ± 0.129	3.125 ± 0.496

Table S8. Calculated q_e (g_{sol}/g_{gel}) values for various gel compositions for 30% DMSO concentration gradient.

Gel Composition	I	II	III	IV	V	VI
Step 1	0.233 ± 0.004	0.231 ± 0.001	0.241 ± 0.005	0.238 ± 0.003	0.237 ± 0.003	0.237 ± 0.003
Step 2	0.197 ± 0.009	0.207 ± 0.001	0.186 ± 0.012	0.213 ± 0.004	0.168 ± 0.003	0.202 ± 0.005
Step 3	0.213 ± 0.003	0.210 ± 0.007	0.150 ± 0.016	0.219 ± 0.003	0.163 ± 0.004	0.211 ± 0.006

Table S9. Calculated k_2 (× 10^2) ($g_{sol}/g_{gel}\cdot min^{-1}$) values for various gel compositions for 30% DMSO concentration gradient.

Gel Composition	I	II	III	IV	V	VI
Step 1	8.617 ± 0.669	8.232 ± 0.437	8.135 ± 0.915	12.990 ± 0.478	7.899 ± 0.294	12.92 ± 0.110
Step 2	7.678 ± 1.129	6.658 ± 0.487	6.545 ± 0.643	12.820 ± 0.751	7.337 ± 1.385	12.870 ± 0.274
Step 3	3.364 ± 0.396	3.775 ± 0.403	3.664 ± 0.672	5.251 ± 0.480	3.657 ± 0.132	5.433 ± 0.549

Table S10. Calculated h (× 10^3) ($g_{sol}/g_{gel}\cdot min^{-1}$) values for various gel compositions for 30% DMSO concentration gradient.

Gel Composition	I	II	III	IV	V	VI
Step 1	4.661 ± 0.211	4.395 ± 0.228	4.728 ± 0.548	7.391 ± 0.357	4.444 ± 0.202	7.249 ± 0.485
Step 2	2.970 ± 0.182	2.858 ± 0.205	2.266 ± 0.232	5.786 ± 0.097	2.082 ± 0.426	5.249 ± 0.262
Step 3	1.521 ± 0.140	1.662 ± 0.082	0.807 ± 0.063	2.531 ± 0.283	0.973 ± 0.022	2.419 ± 0.184

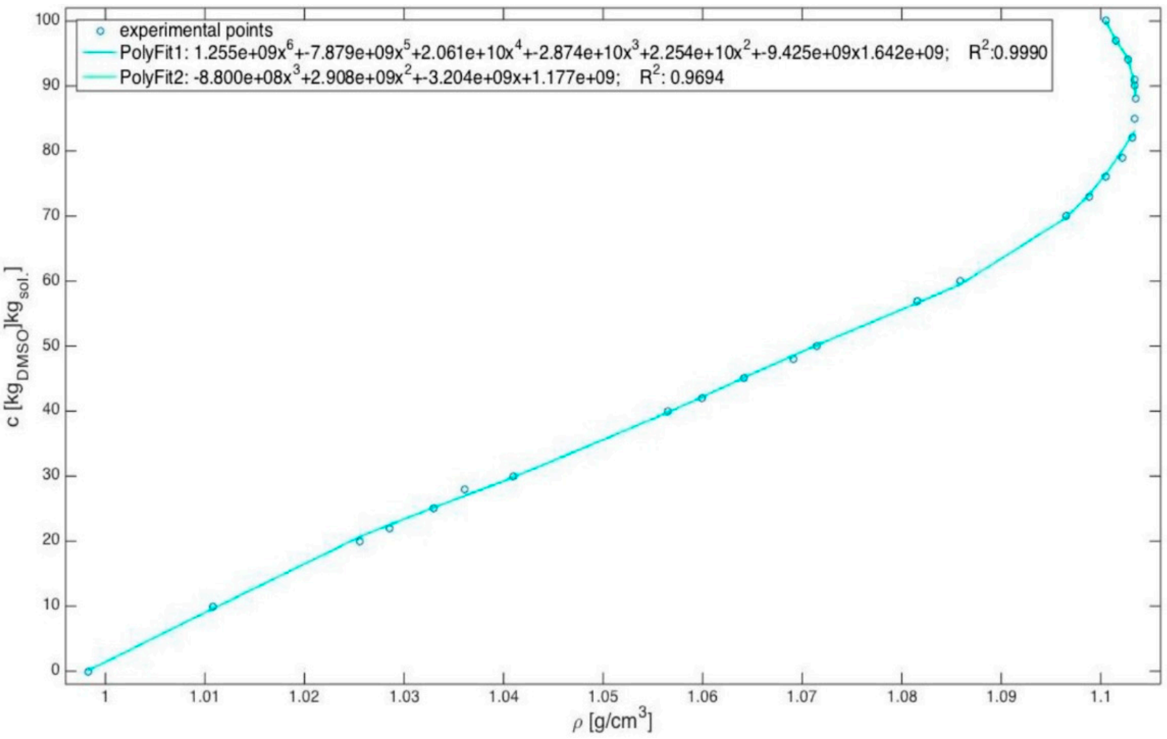

Figure S1. Experimentally determined calibration graph of binary water-DMSO mixture.

© 2015 by the authors; licensee MDPI, Basel, Switzerland. This article is an open access article distributed under the terms and conditions of the Creative Commons Attribution license (http://creativecommons.org/licenses/by/4.0/).
